# Social Capital and Pets in Retirement Villages

**DOI:** 10.3390/ani16111674

**Published:** 2026-05-30

**Authors:** Janette Collier, Virginia Lewis, Pauleen Bennett

**Affiliations:** 1 Australian Institute of Primary Care & Ageing, School of Nursing & Midwifery, La Trobe University, Bundoora, VIC 3083, Australia; v.lewis@latrobe.edu.au; 2 School of Psychology and Public Health, La Trobe University, Bendigo, VIC 3550, Australia; pauleen.bennett@latrobe.edu.au

**Keywords:** pet ownership, older people, retirement village, social capital

## Abstract

Social capital generates collective benefits for a community, making it stronger and more resilient. This qualitative study explored how the inclusion of pets can shape social capital within Australian retirement villages. Semi-structured interviews with village managers and residents identified five themes: perceptions of the impact of pets on (1) owners; (2) other residents; (3) the community; and (4) contextual factors influencing the impact of pets in retirement villages. However, there was an unexpected fifth theme identified around the impact that living in a retirement village could have on pets. Findings indicate that pet-friendly villages can bolster individual well-being and collective social capital. Hence, flexible, adaptable, co-designed policies should include pets as part of the community, as well as incorporating the needs of people who are intolerant of pets for medical, cultural, or personal reasons.

## Simple Summary

Retirement villages want residents to feel involved and part of their community. Pets can improve the well-being of residents and help with community connections. Through a series of interviews with managers and residents of retirement villages, we found that pets can improve the well-being of residents and help with developing connections between them. Having a flexible policy in relation to pets helps both pet owners and non-pet owners alike. It is also important to consider the needs of the pet in the retirement village environment.

## 1. Introduction

Evidence about the impact of pets on the lives of owners is mixed, with some studies showing that pets improve well-being [1,2], while others show no impact on psychological well-being or physical health [3,4]. Less controversially, pets are perceived to play important roles in the lives of their highly attached owners [4], including increasing social connectedness [5,6].

The population of Australia, by June 2024, was approximately 27 million, with 4.7 million (17.3%) who were aged 65+ [7]. This age group is expected to increase to over 8.3 million people by the end of 2050 [8]. Pet ownership rates have recently soared in Australia, reaching 68% in 2022 [9]. Studies have shown that pet ownership rates decline as people age [10,11], with only a small proportion of pet owners aged over 90. If pet ownership rates for people in the younger category of the 65+ age group are half that of those under 65 years, this means that more than 1.7 million people aged 65+ are current pet owners, and that this number will increase markedly in the next decade.

The housing needs of an ageing population are a priority policy issue. Consideration has been given to housing choices for older people with pets wanting to age-in-place or those seeking low-income, emergency, or crisis accommodation [12,13,14]. Research has also been undertaken into the policy and institutional constraints on housing for older pet owners [15,16]. In Australia, nearly 250,000 older people choose to live in one of approximately 3000 retirement villages [17]. These ‘villages’ are typically relatively self-contained communities [18], comprising a group of individual housing structures that may be collectively surrounded by a wall or fence with gated or open access. Within the village, the houses/units may have their own courtyards or no fences between them, and there is usually a central facility that offers shared activity spaces, a kitchen, a gym, or space for visiting services such as hairdressing or allied health. The village may also offer central services such as maintenance or gardening, as well as social activities such as art classes, day trips, or games afternoons [18,19,20]. Regardless of their exact fee structure, which can vary greatly, most retirement villages charge a substantial fee for entry. This makes the decision about which one to relocate to an important milestone in the ageing process.

Older people considering moving to a retirement village are often seeking independent living with lower maintenance, increased security, or social interaction and companionship [18]. A good retirement village is one that provides comfort, security, and a vibrant community [21]. People in a retirement village are clustered together, and social interactions are encouraged by the village operators, who typically provide a community centre for physical or intellectual activities, which are, in the main, organised as social events. These events build connections, trust, and a supportive environment, helping to maintain a happy and healthy lifestyle [22].

Older people living with a pet are likely to consider whether they can retain this pet and how well the pet’s needs can be accommodated as factors in any relocation decision. It has become easier to have pets in rental properties across Australia following changes to residential tenancies laws. These now preclude blanket ‘no pets’ rules, with most states and territories having just a short list of reasons that landlords can use to refuse pets in rental properties [23]. The governance of retirement villages, however, is handled differently [24].

Each jurisdiction in Australia has an individual ‘Retirement Village Act’, with none including a ruling on pets and no direction about whether a policy is required. This allows the operators or, more often, managers (people appointed to manage the village and represent the operators/owners, being responsible for village operations as well as meeting residents’ needs [25]) of each village to set their own rules. In line with broader social changes, more retirement villages are becoming “pet-friendly”, with managers/operators recognising the significance of pets in the lives of older people [16]. There is still a great deal of variability, however, with some retirement villages accepting all pets, some having restrictions on the size or type of pets they will accept, and others accepting no pets at all.

The question underpinning this research is “How do pet policies affect the experiences of people living in retirement villages?” This study examined the views of residents and managers of four retirement villages about the impacts of pets within their village, exploring in particular the contribution of pets to social capital at an individual and community level. Three of the villages had no restrictions on the size or number of pets residents could keep. In the fourth, pets were not allowed.

## 2. Materials and Methods

La Trobe University’s ethics committee approved this study (HEC22289). A target of four or five villages with at least one not accepting pets was set to provide a breadth of experience. Twenty-two organisations were randomly selected from the Property Council of Australia’s ‘A Wise Move’ website [26] and contacted with an invitation to participate. There were 180 retirement village operators listed on the website for Victoria. Of the twenty-two selected, ten organisations operated multiple (2–28) retirement villages, while twelve operated single independent villages. Eighteen villages of those selected had information in the public domain that indicated they were pet-friendly, but four would not allow pets at all. If the initial random sample of twenty-two organisations had not yielded sufficient villages for this study, a further random sample would have been selected from the website.

Contact was made by phone where possible, or email, with follow-up calls and emails made if no response was received. Four single independent villages agreed to participate, sometimes after consulting with a residents’ committee. Three of these said they had no restriction on pets, while the fourth did not accept any pets. This sample size was small, but it did reflect the general situation found during recruitment in relation to acceptance of pets in retirement villages. Two villages were located in metropolitan suburbs, one was on the outskirts of a regional city, and the last village was in a rural area.

Advertisements were sent to each participating village promoting the study and advising where and when focus groups would be held. These were placed in the local village newsletter, which was distributed electronically or on paper. Participant information and consent forms were provided to managers for distribution, but they were not asked to actively recruit participants. Residents who were interested were asked to bring the forms to the session. These signed forms were collected at the beginning of each focus group.

Overall, data were collected from 27 people, including five managers, all of whom were women. Focus groups were held for residents at each village, usually in the central activities room or dining room. Residents were asked to participate in the focus group if they had owned a pet sometime in the year prior to moving into the retirement village or intended to buy a pet once they had moved in. Both men and women voluntarily participated, although more women (*n* = 16) attended than men (*n* = 6). One manager and six residents (one man and five women) attended a focus group or were interviewed from the village that did not accept pets. The lead author met participants for the first time immediately prior to running these groups. Four residents requested an individual interview because they did not want to attend with other people or were away on the day of the focus group. One of these residents, from the non-pet-friendly village, later withdrew before the interview because she reported being too distressed when thinking about her pet situation to continue. One interview was conducted by phone at the participant’s request. All of the residents in this study were retired. Five managers were interviewed in person, three immediately prior to their residents’ focus group and two on a subsequent day. Interviews ranged from 30 min to one hour. Each focus group went for an hour. The lead author has worked as a researcher for more than a decade, so has experience conducting interviews and focus groups.

The questions used to guide focus group and interview discussions are provided in Table 1.

The focus groups and interviews were audio-recorded, and transcripts were produced. Data were analysed using the approach suggested by Bingham [27] for thematic analysis and Nvivo (v15) software [28]. After all transcripts had been read several times, one focus group transcript was coded by the lead author, and initial codes were developed. A second transcript was then coded, and the codes were updated and expanded. This updated list of codes was used to code a third transcript with all authors present. All authors discussed the codes that had been developed so far and modified the codes where needed. Further codes were also added by all authors at this time. All remaining transcripts were then coded by the lead author. The codes were grouped into themes, which were reviewed and reshaped to produce a final outcome that focused on the research question. This meant that some codes were excluded from this paper. The excluded codes included those that covered other non-pet-related aspects of living in a retirement village and pet experiences prior to moving into the village. Reporting of analysis and results was informed by the Consolidated Criteria for Reporting Qualitative Studies (COREQ) checklist [29].

## 3. Results

Five themes were identified in the data and are reported in Table 2. The results are presented below by providing examples of data categorised under each theme.

### 3.1. Theme 1: “The Dogs Are Like My Family”: Impact of Pets on Their Owners

Pets were important to pet-owning residents of the villages, with most of them describing their pet as part of the family:

*But I said to* [my husband], *do they take dogs? Because if it had been no, that would have been it, because the dogs are like my family.*

The companionship provided by the pet was mentioned most frequently:

*And she’s a good companion because I’m on my own. I’ve got someone to talk to.*

Some people mentioned the security that pets provide from other people and other pets.

*…and a lot of women living on their own feel more secure with a dog. I think men do too to some degree.*

*And she’s* [the dog] *walking around and she sees dogs coming around the fence near us; she comes up and just pushes them away so they can’t interfere with us. She’s very protective, very protective.*

Residents also talked about the exercise they obtained with their dogs:

*Yeah, conscious of the fact that having a dog makes you walk. I get so annoyed with people who say, oh you can just take them up there and run them loose. I need exercise too.*

*You see, I wouldn’t walk twice a day if I didn’t have my dog. I certainly wouldn’t walk twice a day. And I have a sore leg, but I make myself do it.*

Some of the residents believed that their pets were good for helping with blood pressure and other elements of physical and emotional well-being:

*If you’re stroking a cat or a dog or a pet* [it] *lowers your blood pressure and all that.*

*And especially with* [my husband] *gone. There’s something alive, you know, in the house that I can talk to and yeah, they’ve been my saviour, my dogs.*

*The presence of an animal in whatever capacity and role. It’s, I think clearly, you know I’ve got a pretty firm view about the benefit to mental health, of having an animal in your life in whatever capacity.*

All of the residents in this study were retired. Another impact of owning a pet was supporting routine and a reason for getting up in the morning:

*So, it’s good for us elderly, whatever, people. To get you more motivated to get out of bed.*

*And the routine, gives us routine. You’ve got to get up even if you don’t want to. I need to feed the cat, the dog or something.*

Life with pets was not perfect, though, with residents acknowledging that there were downsides to owning pets. With reduced incomes for most people once they have retired, costs are an important consideration, with common concerns including pet food, grooming, boarding, and especially vet bills.

*But the expense, I just find it’s got so expensive; it has got so much more expensive, vet bills.*

*…he* [the dog] *suffers from separation anxiety. He’s now developed an itch problem, so I’ve spent a fair amount at the vets already. Yeah, the cat has been to the vet as well.*

Retirement allows more freedom to travel without the tie of employment, but another common impact noted was the restrictions on activity that came from owning a pet. These varied from restrictions in resolving health issues to the ability to go on holidays.

*And I was sick through the year, the doctor kept asking me to go to hospital, I said. No, I can’t leave my cats, he said. Oh, we have got a problem. I said yes. Well, I’d rather die with them, I can’t leave them.*

*And yes, so I am really tied. And I’ve done a lot of travelling and I really would love to still do that. But he’s my priority, so I can’t do it anymore. Now I could not leave him.*

Discussions of age had participants concerned about pets being trip hazards or boisterous. They also considered what would happen if the person had to move into a higher care setting:

*We’ve got someone with a dog, who’s the bigger one and he is only still a puppy. So, he’s quite boisterous. He’s not like a nasty dog, no, but he’s just very friendly.*

*This is like living in high density [accommodation], and it’s older people who have a lot of fall risks.*

*I mean, there are a lot of elderly people in this village and can you imagine them trying to pick up the doggy doos.*

When talking about impacts, managers raised some of the issues that the residents spoke about, including barking dogs, owners not picking up after their pets, and pets being trip hazards.

*… just having to constantly remind people to please follow the rules. But apart from that, I really don’t think there’s a downside. I mean, unless you had someone who wasn’t looking after their pet properly. But we don’t have that here. I mean, they’re babies, you know* [the pets]. *They’re spoilt rotten.*

### 3.2. Theme 2: “I Get My Animal Fix”: Impact of Pets on Other Residents

Residents without pets who attended the focus groups talked about receiving benefits from other people’s pets, without the responsibility:

*I’ve always had dogs and animals. I don’t have one at the moment, but I get my animal fix from* [named people in the village with pets].

*I’ve noticed we’ll do that. They look after each other’s pets or because some people don’t want the responsibility of a dog full time and so they’re more than happy just to have a visit.*

Village managers talked about the general benefits of having pets around for all residents. Residents who did not want the responsibility of owning a pet helped out with walking, minding, petting, or visiting the pets of others:

*We had a lady in with dementia, who didn’t pick up her pet poop. Somebody else picked it up.*

*I’ve taken my dogs down to visit people, and they sit there cuddling them and I’ll go and collect them again, they all get the benefits of that.*

*A couple of the people have brought their dogs* [outside people working in the village]. *I’ve put a bowl out years ago, it’s still out there. But you know, that was nice. You know the workers having the dogs.*

But there was also an acknowledgement that it was not always ideal:

*…you had a group of people there that didn’t like dogs. [the village] had, like, a Facebook group, and I was in that Facebook group and I ended up leaving it, as I just became very intolerant of all the whinging. Oh, someone’s not picking up after their dog and they take photos of it and they post it up […] And I’d say to myself, why do people think that living in a village is different from living out there in the wider community, it’s people. It’s exactly the same. And what you have to tolerate in the community, you need to tolerate here as well. It’s not some controlled artificial environment that you’re actually living in.*

### 3.3. Theme 3: “They Also Connect People”: Impact of Pets on the Community

Participants recognised that pets help with introducing residents to each other in a community.

*They also connect people when you’ve got a pet, you know you can find some common ground.*

*… one of the things I found having [my dog] here is that I got to know everybody because when I arrived with* [my dog], *everyone said Oh hello.*

Two dog owners in different villages had similar concerns about barking impacting their neighbours, but they turned the negative into a positive. One had a dog barking outside at night and, while allowing the barking to commence, had a routine for calming the dog and returning her indoors. Regarding the neighbours, it was stated that:

*… they were grateful. Because they know that if she’s barking, there’s a reason. I’d rather know and be alerted to it than not be aware.*

This dog was responsible for security lights being installed to discourage non-residents from taking a shortcut through the village.

Another had his car broken into at night when parked outside his unit on village property. The dog has become the night security system for the neighbours:

*At night-time, he stays inside, if he does jump off the bed and bark then I know somebody’s around.*

The managers talked about some dogs being boisterous or aggressive to other people and dogs. This was raised as an impact on non-pet-owning residents rather than pet owners. Some participants raised concerns about their pet’s behaviour and impact on their neighbours:

*Our only concern moving into a retirement village is with your dog barking. I worry that she annoys neighbours, but no one has ever complained. But that is a big concern.*

*…we don’t actually walk our dog very much around the village because she gets excited when she sees people coming up and old people can be worried by that.*

### 3.4. Theme 4: “It Depends on Who’s in Charge”: Contextual Factors Influencing Impacts of Pets in Retirement Villages

Managers of the pet-friendly villages all had pets themselves that spent their days with their owners at work. These pet-friendly facilities make it clear to potential residents that pets are a part of village life. One of the managers pointed out the importance of their role in supporting the policy:

*… it’s because I recognise the value of animals. If you’ve got a manager in a village that doesn’t love them [animals], it would go a whole other way. My assistant in the office, she hasn’t had as much training as I’ve had, and her immediate response was, oh,* [the owner]*’s the problem. So yes, it really depends on who’s in charge.*

The manager of the village with a no-pet policy did not own a pet. When asked about the no-pet policy, the manager explained that there were no fences between properties in the village. They also had extensive gardens and issues with foxes and rats. A final concern was that some of the residents, described as potential hoarders, might have a problem with adding animals into that mix.

*…it’s just really hard to manage unless it’s ideal and people are well enough to look after their own animal.*

Participants in the village with a no-pet policy who agreed with the policy were quick to point out issues with having pets, even without being asked:

*This is like living in high density* [accommodation], *and it’s older people who have a lot of fall risks. And there’s hygiene. There’s kitty litter,* [the cats] *would have to be indoors with all the birds. It just makes it, I think, really challenging for the village. And I understand why they say what they say, you know?*

Participants also talked about ways of dealing with the no-pet policy:

*But you sort of learn to live without one, don’t you?*

*Yeah, well, my son’s got cats. So, if you’ve got family and you can visit family … I think that’s a substitute.*

Two of the three villages that allowed pets had formal pet policies; the other was very informal. One village with a formal pet policy had built on a local council initiative to voluntarily register the details of older constituents in case of an emergency. They documented the name, address, and next of kin of residents, as well as details about any pets present, including whether they were friendly to strangers. The village added information about who would care for the pet permanently if the resident could no longer do so, and who would look after the pet in an emergency. Another village only started developing its policy because people were not following the “unwritten rules” by picking up after their pet and keeping it on a lead when out:

*We’ve only really implemented an actual formal pet policy, like that, in the last few years, and that’s only because it kind of become necessary because some people were getting just a little bit disrespectful.*

The pet-friendly village that did not have a formal pet policy expected everyone to “be sensible” about their pets. Those with policies were asked what happened if someone did not follow the rules. In one case, a formal letter was needed as the resident had not responded to conversations. However, all villages said they had never had to go so far as to tell someone to remove their pet from the village. Most of the time, the resident resolved the situation. One manager talked about a couple moving in with a dog who was deaf and howled whenever left alone:

*Fortunately, I didn’t really need to intervene too much; they actually made the decision themselves that they needed to rehome him.*

### 3.5. Theme 5: “She’s Adapted to the Other Dogs”: Impacts on Pets

When asked whether they would have considered their pet when deciding to move into the village, participants talked about looking at the space available before deciding:

*And, also, they even let me bring my cat run which was also good … I wasn’t sure which unit I was going to be looking at, and I’m having one of the old units down the back there and it’s actually got quite a big courtyard. It’s in a nice setting and I thought this would be great for* [my dog]. *Mm-hmm. And I looked at it, I thought, could I fit the cat run in somehow here?*

Although this owner considered their pets, this consideration was mainly about the impact on the owner due to the expense of obtaining new cat runs.

Other pet owners discussed how their pet had to adapt when moving into the retirement village:

*Ohh, she’s never had trouble with other dogs, but well, she’s never really been involved with other dogs like she is here. She meets dogs regularly here, but she’s never lived anywhere where there were dogs around. But since she’s been here, she’s adapted to the other dogs.*

*But the dogs are a little bit on the nervous side since we moved in because everywhere I go, the dog is there and that means bed and everything. It’s part of our life, our little dog.*

*I should have probably had the hindsight because my dog loves to run. I’ll walk her twice a day, but she’s pretty bored all day, so it’s probably not the right place for her really.*

Others talked about helping with the adaptation:

*Make sure they adapt quickly. You can’t just dump them in the yard. Give them the things that make them adapt. Make sure their sleeping things are proper. Dogs have their habits, and their life is one habit after another. And to change that, coming from one environment to another, you’ll have to arrange for them to adapt. That’s important, otherwise you’ll have all sorts of trouble.*

Most of the owners had considered the impact of leaving their pet behind if they died before the pet:

*…the problem with being older is that you’ve got this kind of debate about the benefits of having an animal and then the worry that you know you might* [emphasis added] *outlive them* [or you might not] […] *I worry about mine because I don’t have a family* [who could take care of the animals if something happened to the speaker].

*Like at some point, if you’ve got a pet, you know what I mean, and you go first. That would be sad for me. That would be sadder than having to not have one.*

*That’s why I was getting a middle-aged dog. Because I didn’t, you know, you do think about that* [outliving their pet].

## 4. Discussion

Three themes that were identified in the study data reflected the impact of pets on owners, other residents, and the retirement village community as a whole. The fourth theme related to contextual factors that influenced whether and how pets contributed to life in the village. A final theme reflected the impact on pets of living in a retirement village.

Many of the impacts of pets on their owners who were living in the retirement villages shown in this study are reflected in the general literature about pet ownership, particularly for community-dwelling older adults. In 2021, 10% of the general population of Australia lived alone, and this increased to 25% for those aged 65+, and 32% for those aged 80+ [30]. The first impact identified by both residents and managers was the companionship that the pet provided, reducing loneliness as well as providing support and a presence to talk to. This improves emotional well-being, as found in previous studies with diverse populations [1,2,3,9,31,32,33,34].

Exercise becomes more important to overall health as people age [35], and the participants highlighted exercise and physical well-being as positive impacts of owning a pet. Walking a pet is considered a lower-level activity compared with a brisk walk, and some studies have shown that pet owners do more lower-level activity than non-pet owners [1,9,34], although not all studies reached the same conclusion [5,36]. Other impacts of pets on owners reported in this study included providing a routine, a reason to get out of bed in the morning, and a way of easily connecting with others. Residents, particularly dog owners, said their dog encouraged conversations while walking. This is also reflected in the literature [2,5,9,33,34].

The interviewees also acknowledged that there were negative impacts associated with pet ownership: financial strain, restrictions on travel, grief and loss, fear of outliving the pet, and possible trip hazards. Again, these are reflected in the existing literature about the consequences of owning a pet [9,32,33,37]. Most of these negative impacts were reported by pet owners who all said they would not be without their pet; therefore, the positive impacts are obviously greater.

Managers were more likely to look at the bigger picture and recognise the positive and negative impacts of pets on the wider village community. These included the benefits of being able to talk to and touch a pet without the associated responsibility, and the negatives associated with residents not picking up after a pet, as well as barking, boisterous, or aggressive pets.

The themes identified in the data suggest that pet ownership in retirement villages is contributing to social capital. Putnam [38] (p. 19) describes social capital as referring to “connections among individuals—social networks and the norms of reciprocity and trustworthiness that arise from them”. Others have explored social capital on an individual as well as a community basis. At the individual level, social capital provides rewards for each person involved in building networks and trust. It could be favours between neighbours, such as putting bins out or feeding a pet. At the community level, social capital extends beyond the individual and instead provides collective benefits, regardless of whether all individuals play a role in building connections [20], making a neighbourhood more secure, for example.

Social capital theory posits that increasing connections and trust are critical for improved development and cooperation. This has significant implications within the retirement village context. The contribution of pet ownership to social capital and the nature of social capital in retirement villages have been considered independently at both individual and community levels [20,39,40]; however, the contribution of pets to social capital in retirement villages and the potential impact that this has on housing choices of older people has not been explored.

Pets build social capital at both individual and community levels. Walking a pet can initiate conversations with pet owners and non-pet owners alike. These social interactions are at the heart of developing individual social capital [40]. However, regular pet walking also provides opportunities for walkers to keep an eye on the neighbourhood and may improve the security of the area [41]. Dogs in the workplace have also been shown to improve the corporate atmosphere and build social cohesion among employees [42]. The contribution of this study is to demonstrate that these same benefits are recognised by retirement village residents, with participants strongly endorsing that their pets contribute to the development of the community.

A retirement village wants to develop social capital—the more social capital they develop, the more successful the retirement village will be [20]. Pets can clearly help with this by contributing to a ‘sense of community’ for pet owners and non-pet owners, facilitated through increased social engagement and an increase in safety brought about by more people walking the village streets with their dogs [43]. Pets, by enhancing social interactions, also improve quality of life and social connectedness [5]. Even negatives can become positives, with this study finding that a pet that barked led to improvements in retirement village security. Pet ownership, therefore, improved social capital for the village at the community level as well as for owners and non-owners at the individual level.

Two-thirds of people in Australia currently have pets, and 60% of households without a pet are actively interested in acquiring one [9]. With an anticipated population increase in the number of people aged 65+, more people will want to enter retirement villages and more will want to take their pets with them. As reported earlier, not all retirement villages are pet-friendly. For the non-pet village in this study, reasons provided were a lack of fences between properties, extensive gardens, and a chance of residents hoarding pets. These are all issues that could be managed. Millions of dogs worldwide live in places without fences, such as apartments, as described by Power [44], and keeping pets on leads while in common areas could ensure that gardens are not destroyed. To combat hoarding, management could require residents to register their pets, including providing plans for emergency care if required, as modelled by one pet-friendly village in this study. Other issues may be more difficult to manage, including providing pet-free spaces for residents who dislike animals or who need to avoid them for cultural or medical reasons. These issues are not specific to retirement villages, however, so effective management strategies used in the broader community may be applicable.

Are formal policies necessary? Having a formal policy implies that someone will enforce behaviour where the rules are not upheld, and some social capital advocates would argue that enforcement is the opposite approach to building social capital [38,45]. Clark and McCann [22] (p. 164), for example, argue that formal policies are not needed to regulate behaviour as “these features of social capital are all underpinned by shared social norms governing the behaviours that are appropriate or acceptable in a given setting”. Some of the interviewees referred to this type of control as showing “respectfulness” and commented that they only intervened in a potentially negative situation when behaviour did not appear to be guided by social norms. Other authors, however, have extended the definition of social capital to include sanctions, which can be informal or formal, direct or indirect [46]. Imposing sanctions requires that expectations be clearly laid out in a formal policy.

That pets can be managed effectively in retirement villages was shown by three of the four villages in this study. Two had well-developed pet policies. The third did not have a formal pet policy; however, management simply expected the community to adhere to social norms. This village was smaller and did have informal policies reflecting good pet ownership behaviour in place—the ‘social norms’ of social capital. In this study, neither formal nor informal pet policies needed much enforcement, with managers reporting that most residents self-monitored their pet’s behaviour as well as their own. One formal letter had been sent to a resident requesting a change in their behaviour, but no resident or pet had been removed from a village by policy enforcement. Furthermore, effective communication, both clear and open, is crucial for a successful pet-friendly policy, no matter the setting [42,47].

This was a small study that used qualitative methods to explore the research question through the perspectives of a small set of participants. This study included some individual interviews at the residents’ request, which is more likely to be impacted by the interviewers’ views than a focus group. One interview was conducted by phone, which may have reduced the rapport generated between the interviewer and participant. It is also important to acknowledge the positionality of the researcher in qualitative research and the impact this may have. In this instance, all authors have favourable views about the benefits of pet ownership for older people.

## 5. Conclusions

There are cultural and personal differences in attitudes to pets; not all people like or want to live with pets. However, this should not prevent retirement villages from becoming pet-friendly; pets are not banned from general communities for these reasons. Pets contribute to the social capital of the village and of the individuals who encounter them. Pet-friendly policies should be able to address any potential concerns. Any pet-friendly policy needs to be flexible and adaptable. The needs of pets should also be considered; for example, offering secure off-leash areas in the village where pets and their owners can socialise, or a space within the central facility where indoor pet-related activities such as training and pet treat-making classes could occur. Indoor no-pet spaces should also be established so that those with pet-related phobias or allergies are not excluded from the social life of the village.

For new retirement villages, starting with a village that is pet-friendly is more straightforward, as rules and spaces can be developed before residents move in. For existing no-pet villages, a good option would be to co-design the rules with the residents, including those who do not have or do not like pets.

## Figures and Tables

**Table 1:** Manager and resident questions.

Manager	Resident
Can you tell me a bit about your background?	Can you tell me a bit about your background?
What do you do here in the village?	Have you had pets in the past? What about now?
Have you had pets in the past? What about now?	What sort of place were you looking for when you moved in here?
Can you tell me about the pet policy here?	Were you planning on taking your pet with you?
How long has that been the policy?	Did you talk to the village about your pet before deciding to move in?
Does the nature of the policy have any impact on the residents?	What would it have meant if you couldn’t move with your pet?
What are the positives about the pet policy?	What are the benefits of having pets?
What about negatives?	What about downsides of pet ownership?
How do you feel about pets in the village?	Any advice for others considering moving into a retirement village?

**Table 2:** Themes identified during analysis.

Theme
Impact of pets on their owners
Impact of pets on other residents
Impact of pets on the community
Contextual factors influencing the impact of pets in retirement villages
Impact on pets

## Data Availability

Many of the original contributions presented in this study are included in the article. Further inquiries can be directed to the corresponding author. The dataset is not available publicly to protect the privacy of the people interviewed.
